# Identification of barriers and facilitators for optimal cesarean section care: perspective of professionals

**DOI:** 10.1186/s12884-017-1416-3

**Published:** 2017-07-14

**Authors:** Sonja Melman, Rachel Hellen Petra Schreurs, Carmen Desiree Dirksen, Anneke Kwee, Jan Gerrit Nijhuis, Nicol Anna Cornelia Smeets, Hubertina Catharina Johanna Scheepers, Rosella Petronella Maria Gemma Hermens

**Affiliations:** 1 Department of Obstetrics and Gynaecology, GROW- School for Oncology and Developmental Biology, Maastricht University Medical Centre, Maastricht, The Netherlands; 2 Department of Obstetrics and Gynaecology, Zuyderland Medical Centre, Heerlen, The Netherlands; 3 Department of Clinical Epidemiology and Medical Technology Assessment (KEMTA), CAPHRI- School for Public Health and Primary Care, Maastricht University Medical Centre, Maastricht, The Netherlands; 4 Department of Obstetrics and Gynaecology, University Medical Hospital Utrecht, Utrecht, The Netherlands; 5 Scientific Institute for Quality of Healthcare (IQ healthcare), Radboud University Nijmegen Medical Centre, Nijmegen, The Netherlands

**Keywords:** Barriers, Facilitators, Guidelines, Cesarean section, Professionals

## Abstract

**Background:**

The cesarean section (CS) rate has increased over recent decades with poor guideline adherence as a possible cause. The objective of this study was to explore barriers and facilitators for delivering optimal care as described in clinical practice guidelines.

**Methods:**

Key recommendations from evidence-based guidelines were used as a base to explore barriers and facilitators for delivering optimal CS care in The Netherlands. Both focus group and telephone interviews among 29 different obstetrical professionals were performed. Transcripts from the interviews were analysed. Barriers and facilitators were identified and categorised in six domains according to the framework developed by Grol: the guideline recommendations (I), the professional (II), the patient (III), the social context (IV), the organizational context (V) and the financial/legislation context (VI).

**Results:**

Most barriers were found in the professional and organizational domain. Barriers mentioned by healthcare professionals were disagreement with specific guideline recommendations, and hesitation to allow women to be part of the decision making process. Other barriers are lack of adequately trained personal staff, lack of collaboration between professionals, and lack of technical equipment.

**Conclusions:**

Clear facilitators and barriers for guideline adherence were identified in all domains. Several barriers may be addressed by using decision aids on mode of birth or prediction models to individualise care in women in whom both planned vaginal birth and CS are equal options. In women with an intended vaginal birth, adequate staffing and the availability of both fetal blood sampling and epidural analgesia are important.

**Electronic supplementary material:**

The online version of this article (doi:10.1186/s12884-017-1416-3) contains supplementary material, which is available to authorized users.

## Background

The cesarean section (CS) rate has increased over recent decades in both developed and developing countries. The World Health Organization has targeted the CS rate to be 10% to 15%, although this low rate has recently been questioned [1, 2]. In The Netherlands, the CS rate has increased steadily over the past decade from 10.8% in 1999 to 16.5% in 2014 [3, 4]. In absolute numbers, the most impressive rise was documented among healthy women with a singleton in vertex position between 37 and 42 weeks gestation [5]. Furthermore, in The Netherlands in 2013, a large variation in vaginal birth rates per hospital were observed in the nulliparous term singleton vertex group, with rates ranging from 45.1–73.2% [6].

This worldwide increasing CS rate is a cause for concern since this will lead to increased maternal and neonatal complications, increased risks for future pregnancies, and increased healthcare costs [7–10]. Literature mainly focusses on individual-level explanations for the CS rise, including increased maternal age, technological innovation, women’s choice or clinical risk factors such as obesity and previous CS. However, these explanations do not account for the majority of the variation observed [11].

In order to optimize CS practice, the Royal College of Obstetricians and Gynecologists (RCOG) developed an evidence-based guideline with clear recommendations that have a direct effect on the decision to perform a CS [12]. In the CS implementation study (SIMPLE), a RAND-modified Delphi procedure resulted in 27 quality indicators describing optimal CS care based on international guidelines and literature [13]. The adherence to CS quality indicators was measured in 21 hospitals in The Netherlands in order to gain insight into current obstetrical care.

Besides insight into optimal and current care, insight into facilitators and barriers among all professionals involved in CS decision-making is essential. This insight can support the development of a tailored strategy to overcome barriers and improve guideline implementation. The present qualitative study explored facilitators and barriers for optimal CS care among midwives, obstetrical residents and obstetricians.

## Methods

### Design

A qualitative study was conducted to determine facilitators and barriers that influence the decision to perform a CS from a healthcare professional’s perspective. Two separate focus group interviews were performed; one with obstetricians and one with obstetrical residents from different regions and different types of hospitals in The Netherlands. Due to organizational difficulties, a focus group among independent midwives could not be arranged. Therefore, we conducted telephone interviews among nine midwives until data saturation was reached (the last interview did not reveal any new information).

### Setting

Currently in The Netherlands, pregnant women without medical complications are supervised by a registered midwife (primary care) and have the possibility to choose where to give birth (at home or in an outpatient hospital setting). If medical complications exist or develop during pregnancy or birth, the attending midwife refers the pregnant woman to an obstetrician in a hospital (secondary care). Certain obstetric situations may lead to the advice to deliver by planned CS, whereas others may lead to the need for counselling on risks and benefits of a planned vaginal birth opposed to a planned CS. Mode of birth counselling is mostly performed by obstetricians and residents. Therefore, all situations that may lead to a planned CS are the responsibility of secondary care. However, referring midwives may provide their opinion before referral and are often consulted by the women for a second opinion and are therefore an influencing factor.

### Study population

Thirty obstetricians and residents from university and non-university hospitals in different regions of The Netherlands were invited to participate in focus group interviews. The obstetricians and residents were all members of the Dutch Society of Obstetrics and Gynaecology (NVOG). The midwives were invited based on recommendations from the Royal Dutch National Association of Midwifery (KNOV) and worked in primary or secondary care.

### Development interview guide and data collection

Quality indicators from the SIMPLE study were used as a base for exploration of barriers and facilitators (Table 1) [13].

**Table 1:** Quality indicators in caesarean section care

1) Quality indicators on planned CS
*a) General counseling, CS is not mentioned (vaginal birth is the normal conduct)*
1. Twin pregnancy and first child cephalic position
2. Fetal macrosomia (<4.5 kg in maternal diabetes, <5 kg no maternal diabetes)
3. Preterm labour, cephalic position
4. Small for gestational age without fetal distress
5. Previous shoulder dystocia without impaired perinatal outcome
*b) Counseling directed at vaginal birth (vaginal birth and CS are options, vaginal birth is preferred)*
6. Position of the placenta at 1-2 cm of the internal os
Request for CS without medical grounds:
7. Explore reason for request
8. Discuss (dis)advantages to CS birth
9. In case of extreme fear: offer psychological counselling
10. Preterm breech birth (frank, complete breech)
*c) Counseling mentioning both vaginal birth and CS as equal options*
11. Breech presentation at term
Previous CS (inform on risks and chance of successful vaginal birth after cesarean)
12. Inform on low risk of uterine rupture
13. Inform on high chance of successful vaginal birth after cesarean
14. Inform on increased risk and lower success rate in case of need for labour induction
*d) Prevention of planned CS*
15. Offer external cephalic version in case of non-cephalic position
16. Use of internal audit on CS
2) Quality indicators on emergency CS
17. In case of suspected fetal distress use ST analysis or micro blood analysis
In case of non-progressive labour first stage:
18. Rupture of membranes,
19. Urinary catheterization,
20. Use of pain medication, preferably epidural analgesia,
21. Adequate contractions or augmentation of labour
In case of non-progressive labour second stage in nulliparous women:
22. Active pushing recommended,
23. Adequate contractions recommended,
24. Consider vacuum extraction if the head is <1/5th palpable per
Abdomen
25. Continuous support during labour for women with or without prior training
26. Use of partogram
27. Involvement of consultant obstetrician in decision making for CS

These quality indicators were based on key recommendations on CS care, abstracted from guidelines of the RCOG, the American Congress of Obstetricians and Gynaecologists, the Dutch Society of Obstetrics and Gynaecology and the Society of Obstetricians and Gynaecologists of Canada. The indicators considered mode of birth counselling, planned CS and prevention of (emergency) CS, and were discussed separately during the interviews. Facilitators and barriers were explored at different domains, based on the model developed by Grol [14, 15] concerning the guideline recommendations itself (domain I), the professional (domain II), the patient (domain III), the social domain (domain IV), the organizational domain (domain V) and the financial/legislation domain (domain VI). The focus group interview among obstetricians was moderated by a project member (obstetrician). The focus group interview among residents was moderated by another project member (resident). Individual telephone interviews among midwives were conducted by the same resident.

### Data analysis

The interviews were fully transcribed and analysed using the qualitative analysis tool of ATLAS.ti GmbH Version 7 (Berlin, Germany). Two reviewers (SM and RS) independently read the interviews and coded facilitators and barriers for each of the recommendations and transcripts. The identified factors were assigned to the appropriate domains. Disagreements were resolved by discussion between the reviewers. If there was no consensus, a third independent reviewer was consulted (RH).

## Results

The obstetricians worked at non-university non-teaching hospitals (*N* = 6) or non-university teaching hospitals (*N* = 4). Five residents from a non-university hospital and five residents from a university hospital were included. The midwives worked either in a private practice (*N* = 5) (rural and urban areas), non-university hospital (*N* = 3) or university hospital (*N* = 2).

The quotations were categorised, resulting in a total of 38 barriers and 11 facilitators which mainly concerned the professional and organizational domain. These are described in detail hereinafter (Table 2).

**Table 2:** Professionals’ perceived barriers and facilitators for adherence to CS guidelines

Most important influencing factors per domain:				
Domain I Guideline itself	Domain II Professionals	Domain II Professionals	Domain IV Social	Domain V Organizational
Design • The guidelines are designed for an average patient, instead of an individual	Clinical characteristics • Non-adherence to the guidelines due to patient characteristics	Information by others • Negative experience of friends/family (eg with external cephalic version)	Hampering collaboration • Between obstetricians and anesthesiologists regarding epidural analgesia • Between midwives and obstetricians/residents	No agreements regarding • Responsibility for counseling: the midwife or obstetrician • Variation in standard policy between hospitals (regarding e.g. fetal scalp blood sampling, breech deliveries) • Indications for CS
Availability • Guidelines are not easy available • Local protocols are not up to date	Counseling • No or too late agreement regarding the possibility of a preterm birth	Patients’ view • Patients do not accept any risks for the fetus. • Refusal of external cephalic version.	Variation in policy • Between different obstetricians	
	Documentation • Unclear documentation on mode of birth counselling • Unclear documentation on previous births		Strict hierarchy • Preventing adequate feedback	Staffing • Appropriate staff not present at the discussion regarding obstetrical decisions • Inadequate staffing and allocation of tasks • Midwives are not invited for audits
	Knowledge and skills Insufficient experience or expertise regarding: • Breech deliveries • Foley catheter induction in case of previous CS • Estimation of fetal weight • Fetal scalp blood sampling • External cephalic version			Availability of staff • Obstetricians • Anesthesiologists • Inadequate to provide 1-on- 1 support to women in labour
	Attitude • Policy depends on time (day/night) • Fetal scalp blood sampling is time consuming			Availability of diagnostics • Partogram • Fetal scalp blood sampling
	Disagreement with guidelines • Behaviour therapy in case of fear for pain is not always strictly necessary • Epidural analgesia is not strictly necessary in case of failure to progress in labor • Don’t use a partogram in case of rapid labour progression ▪ CS should be mentioned in case of severe shoulder dystocia in previous pregnancies, even if there is no residual damage ▪ Assessment by an obstetrician in case of failure to progress before performing a CS depends on a residents’ experience			

### Domain I: The CS guideline recommendations

All types of professionals mentioned that guidelines do not adequately consider individual patient characteristics that might influence guideline adherence. As one of the obstetricians described:

*‘If the woman is nulliparous, pregnant with a child that is expected to be large for gestational age and with a fetal head not engaged at term, it depends on her characteristics whether or not I will discuss a CS’.*

This barrier mainly applies to guidelines aiming at vaginal birth in specific situations, such as fetal macrosomia, labour dystocia, breech presentation and previous shoulder dystocia. Furthermore, obstetricians mentioned that guidelines were not easily available on the professional website, whereas residents noted that local protocols were not always recently updated.

Furthermore, not all professionals agreed with guideline recommendations. Almost all types of professionals disagreed with the recommendation: ‘A CS should not be mentioned in case of a previously experienced shoulder dystocia if there is no residual neonatal damage’. The decision to offer a CS mainly depends on the severity of the previous shoulder dystocia, and not on residual neonatal damage alone. A midwife describes:

*‘It depends on the client whether or not I would discuss a CS or induction of labour. If the shoulder dystocia was severe, I would not risk to experience that again.’*

Domain II: The professional domain

Obstetricians and residents mentioned unclear documentation on previous deliveries, including advice on mode of birth in future pregnancies, as an important barrier for guideline adherence. Furthermore, they stated that incomplete counselling or unclear documentation of the decision making process for mode of birth in the current pregnancy, is an important factor for acting in line with the agreed mode of birth. For example:

*You have planned a CS for a woman with a history of fetal macrosomia (4500 grams) and shoulder dystocia. If labour starts prematurely, you might advise her to undergo a vaginal birth, since the expected fetal weight is probably less than 4000 grams’.*

Lack of experience regarding clinical skills in daily practice was also mentioned among all types of professionals to affect guideline adherence, mainly concerning vaginal breech deliveries, fetal scalp blood sampling and external cephalic version. A midwife adds:

*‘The mode of delivery in case of a breech presentation depends on the expertise of the obstetrician in attendance’.*

One of the obstetricians describes:

*‘I believe that we perform a fetal scalp blood sampling about 5 times a year’…. ‘I think this is probably due to insufficient expertise among some of the obstetricians’.*

Another barrier mentioned by the residents regarding the use of fetal scalp blood sampling is the large variation in policy between obstetricians as well as between hospitals. Some obstetricians favour a vacuum extraction when full dilatation of the cervix is reached, whereas others would perform fetal scalp blood sampling in order to, possibly, avoid an operational vaginal birth.

Obstetricians mentioned that the ability to evaluate a cardiotocogram during delivery on any computer as facilitator for optimal care. This improves communication between the obstetrician on call and the resident without the necessity to be present at the delivery ward.

### Domain III: The patient context

A barrier mentioned by obstetricians and residents is mode of birth advice by friends or family of a pregnant woman. Particularly negative experiences regarding the outcome of the neonate are perceived to be cause of anxiety and reduced cooperation of a pregnant woman, which can complicate communication between a pregnant woman and her caregiver. An example: *‘You can never ignore the information a patient receives from a neighbour or a niece. That sometimes seems more important than the medical information you provide.’*

Another barrier mentioned by obstetricians is that they feel it might be difficult for women to adequately balance current and future fetal risks, next to maternal risks and benefits in choosing mode of birth.

*‘Counselling in pregnancy is fundamentally different, since it concerns the mother but also her child. Women seem more concerned for their current child than for a possible future pregnancy. Sometimes weighing the risks of a vaginal birth after previous CS and taking consequences for future pregnancies into account, is difficult even for healthcare providers’.*

Domain IV: The social context

All types of professionals mentioned troublesome collaboration as a barrier. Some residents might hesitate to call the anaesthesiologist to provide epidural anaesthesia, especially at night. This might reduce the number of women with adequate pain relief. As one of the residents described:

*‘In our hospital, the residents are not allowed to independently consult the anaesthesiologist at night. We first have to call the obstetrician, and he or she has to consult the anaesthesiologist. It is a barrier for providing epidural anaesthesia at night.’*

Clear agreements on availability of epidural analgesia are mentioned to be a facilitator in this situation.

The residents and midwives mentioned the presence of a strict hierarchy and wide variation in obstetrical policy between obstetricians as barriers for collaboration. The hierarchy causes some residents and midwives to be reluctant in providing feedback on a decision. An open attitude is essential in an obstetrical team. Residents add that the decision to perform a CS for non-progressing labour might also depend on the time (evening or night).

### Domain V: The organizational context

Some barriers are specifically related to the Dutch obstetrical care system. All types of professionals mentioned there are no general agreements on the responsibility for mode of birth counselling. Some obstetricians feel they should be responsible for mode of birth counselling. One of the midwives described:

‘*Obstetricians are sometimes even angry that we already performed the mode of birth counselling and that the pregnant woman chose her mode of birth..’*

In women with a previous CS, obstetricians and residents stated that, although a consultation in secondary care is preferred before 20 weeks of pregnancy, the current habit of referral to secondary care at 36 weeks of pregnancy seems too late for an adequate shared decision making process on mode of birth. Women are often already fully focussed on a particular mode of birth, which makes counselling by an obstetrician more difficult. Protocols for cooperation between first and secondary care, considering mode of birth counselling, as well as timing of referral, would facilitate optimal care.

Considering the use of additional diagnostics for evaluating the fetal condition during labour, prior to performing a CS for suspected fetal distress, there are several barriers perceived by residents and midwives. Fetal scalp blood sampling might be limited due to technical limitations during sample collection or sample assessment in laboratories. The procedure seems time consuming and often needs to be repeated. One of the residents adds:

*‘At first, we had to send the collected blood samples to the laboratory. After they bought a new device, approximately 3 out of 4 samples could not be analysed. You do not wish to experience that.’*

Domain VI: The financial/ legislation domain

Finally, almost all professionals mentioned that there is insufficient staffing and monetary compensation in order to provide continuous support to women during labour. One of the obstetricians mentions:

*‘In our hospital improved support during labour could reduce CS rates. However, we know upfront that an increase in staffing is not an option.’*

## Discussion

### Main findings

In order to improve CS care, and possibly lower the rate of unnecessary CS, a high level of adherence to evidence based clinical guidelines is required. We conducted a qualitative study to determine which factors influence the adherence to recommendations that have a direct effect on the decision to perform a CS. Among professionals, we identified 11 main facilitators and 38 main barriers for optimal care to previously developed quality indicators on mode of birth counselling, planned CS and prevention of (emergency) CS [13].

In our previous study on actual care, four groups of women with highest expected impact on improvement of care were identified: 1.women with a previous CS, 2. nulliparous women without continuous support during labour, 3.nulliparous women with an unplanned CS performed for suspected fetal distress without applying additional diagnostics (ST-analysis or fetal scalp blood sampling), and 4. nulliparous women with an unplanned CS performed for non-progressing labour without adequate waiting period (2–4 h) [13]. The barriers and facilitators will be discussed with regard to these target groups in order to apply these factors in an implementation strategy.

Regarding counselling women with a previous CS, professionals hesitate to allow women to be part of the decision making process, since they fear women are not equipped to decide on mode of birth when balancing risks and benefits for both their own health, that of their baby and a possible future pregnancy. A main facilitator for obstetricians and residents was detailed mode of birth counselling and clear documentation of the decision making process for mode of birth. This emphasizes the importance of adequately describing the counselling process and also to discuss exceptions or conditions to the agreed mode of birth. Structured counselling using decision aids can help to address women’s anxiety. When the content of this counselling is agreed upon by both first and second line healthcare providers, variation in care between different healthcare providers is likely to reduce. The use of prediction models may help to individualise care.

Insufficient staffing due to lack of monetary compensation is mentioned as the main barrier to provide continuous support to women during labour. Although the Dutch ministry of Health encourages continuous support during labour, this is not realized in the majority of cases.

The main barriers mentioned for applying additional diagnostics prior to performing a CS for suspected fetal distress were a lack of technical skills, but also technical limitations during sample collection or sample assessment in laboratories. A structured training programme might improve care and technical skills. Furthermore, the failure rate during sample assessment might improve with the introduction of fetal scalp lactate sampling [16].

Several barriers and facilitators were mentioned regarding nulliparous women with an unplanned CS performed for non-progressing labour. For example, 24/7 availability of epidural analgesia is agreed upon, however this is not always implemented. Clear agreements on availability of epidural analgesia is mentioned to be a facilitator and might improve the number of women with adequate pain relief. The remarks stating that timing of a CS is partially based on the time of day suggests that availability of different healthcare providers is a clear barrier.

### Interpretation

The present study is, to our knowledge, the first to analyse facilitators and barriers for optimal CS care with international guidelines as a basis. Chaillet et al. studied the implementation of guidelines on vaginal birth after CS in Quebec, Canada. They concluded that adoption of guidelines may be improved if local healthcare professionals’ perceptions are considered [17]. In line with our study, others identified availability of equipment and staff, skill levels, acceptance of guidelines and women’s motivations to be of influence [17, 18].

There are however, some factors that can be explained by cultural differences or local habits, for example economical, political or medico-legal concerns. In the study by Chaillet et al., obstetricians mentioned medico-legal concerns on several occasions as a barrier for optimal care [16]. This is not in line with our findings, probably due to differences in medico-legal habits. The study of Yazdizadeh et al., revealed several barriers regarding the economic and political domain, which may be explained by a differently regulated healthcare system in Iran [18].

The unique structure of the Dutch obstetrical care system entails several challenges for health care providers in terms of continuity of care, referral of pregnant women and responsibility of care. When considering the Dutch healthcare system, improved collaboration between midwives and obstetricians seems an important step in improvement of care.

Although evidence based guidelines are the basis of our obstetric management, the obstetrical healthcare professional is faced with multiple factors in a single patient. There is no clear protocol for a woman with multiple problems during birth, for example macrosomia and a prior CS in combination with suspected fetal distress during labour. That is starting point for discussing the concept of mindlines, first described by Gabbay et al. Mindlines are collectively reinforced, internalised tacid guidelines. These guidelines are based on personal experience, experience of colleagues, as well as interactions with colleagues, opinion leaders and patients and are often developed in early training. These mindlines can be modified e.g. based on brief reading or interaction with colleagues and individual patients. Protocols that are easily accessible as well as up to date are required to initially set and maintain mindlines. In order to change mindlines and keep them up to date, (local) opinion leaders might play an important role [19, 20]. In the current study we did not evaluate these mindlines. However, when considering CS in complex cases, one can imagine the importance of CS audits. An audit can promote the development and modification of mindlines, based on the discussion with colleagues.

### Strengths and limitations

As framework for the interviews (Additional file 1), we used recommendations derived from evidence based guidelines on CS care, as described in the SIMPLE study [13]. Barriers and facilitators were explored for those factors that are considered as most important for measuring optimal CS care.

We invited different types of obstetric healthcare providers that might have an influence on the offered CS care to participate to this qualitative study: obstetricians, residents and midwives, respectively. Professionals from different types of hospitals and private practices from different Dutch regions participated to this study, thereby representing all different elements of Dutch obstetrical care. The different types of healthcare providers were interviewed separately, in order to let them speak without restraint. The main barriers and facilitators were frequently mentioned among all different types of professionals, suggesting that the identified barriers are useful for the development of a new implementation strategy.

Although a standardized method for the identification of barriers and facilitators was used, there are some limitations to this study. First, the analysis of the interviews is prone to interpretation bias. In order to reduce this bias, all interviews were interpreted by two independent reviewers, based on the theoretical model by Grol [14, 15].

Since the influencing factors were identified using a qualitative, explorative method, no quantitative conclusions can be justified by this method. Finally, this study is performed in a national setting. The interviews were based on previously developed quality indicators, derived from national and international guidelines. We identified some facilitators and barriers that are typical for the Dutch system, concerning the collaboration between first and secondary care: for example the responsibility for mode of birth counselling and barriers relating to the timing of referral from midwife to obstetrician. It is, however, in line with literature that there are barriers specific to regional habits and practice [17, 18]. With international guidelines as a basis, we consider most results to be potentially relevant for international use, since our results describe the availability of equipment and staff, acceptance of guidelines and women’s motivations to be of influence in line with literature.

## Conclusions

In light of the rising CS rate, clear facilitators and barriers for guideline adherence were identified at all domains, yet particularly on professional and organizational domain. Professionals should be aware of the identified barriers and facilitators and take these into account when implementing CS guidelines into daily practice.

## Additional file

Additional file 1: Interview guide. Description of the framework of the interviews. (DOC 42 kb)

## Abbreviations

CS: Cesarean section
KNOV: Royal Dutch National Association of Midwifery
NVOG: Dutch Society of Obstetrics and Gynaecology
RCOG: Royal College of Obstetricians and Gynecologists
SIMPLE: Caesarean Section IMPLEmentation study
