# Supplementary material for: Identification of barriers and facilitators for optimal cesarean section care: perspective of professionals
Source: BMC Pregnancy Childbirth. 2017 Jul 14;17:230. doi: 10.1186/s12884-017-1416-3 (PMC5513406; doi:10.1186/s12884-017-1416-3)
Supplement: Additional file 1: — Interview guide. Description of the framework of the interviews. (DOC 42 kb) [file 12884_2017_1416_MOESM1_ESM.doc]

##### Interview guide SIMPLE

**Design:**

General information and background

Introduction of participants

Discussion of barriers and facilitators per recommendation

Conclusion

# Objective: analysis of barriers and facilitators for CS guideline adherence.

Set of CS recommendations developed by professionals according to the RAND-modified Delphi method.

**Introduction:**

Welcome. The SIMPLE study is a research project on CS guideline adherence and when to perform a CS. A set of recommendations based of national and international guidelines was previously developed by an expert panel. The adherence to CS guidelines was then measured to gain insight into current care.

The objective of this interview is to gain insight into barriers and facilitators for guideline adherence. Barriers can exist at different levels: the guideline itself, the professionals, the patient, the social context, the organizational context and the financial/legislation context.

This interview will be recorded, the information will used anonimously.

#### Introduction of participants

**Possible barriers/ facilitators to guideline adherence in general:**

- Are the guidelines easily accessible?
- Are the national guidelines used as a basis for local protocols?
- Are the guideline recommendations easy to use, clearly described?
- Is it a disadvantage that no general CS guideline exists?

#### **Discussion of CS recommendations:**

#### 1. Planned CS:

1. Situations were general counselling on mode of delivery is advised: CS is not mentioned, VD is the normal conduct.
2. Situations were counseling is directed at VD: VD and CS are options, VD is preferred.
3. Situations were VD and CS are mentioned as equal options.

2. Emergency CS

3. Prevention of CS

**Situations to be discussed:**

1a. Twin pregnancy and first child in cephalic position

Fetal macrosomia (<4.5 kg in maternal diabetes, <5kg no maternal diabetes)

Preterm labour, fetus in cephalic position

Small for gestational age without fetal distresss

Previous shoulder dystocia without impaired perinatal outcome

1b. Position of the placenta at 1-2cm of the internal os

Request for CS without medical grounds:

Explore reason for request

Discuss (dis)advantages to CS delivery

In case of extreme fear: offer psychological counseling

Preterm breech delivery (frank, complete breech)

1c. Breech presentation at term

Previous CS (inform on risks and chance for succesful VBAC)

Inform on low risk of uterine rupture (<1%)

Inform on high chance of successful VBAC (overall 75%)

Inform on increased risk and lower success rate in case of need for labor

induction

# 2. In case of suspected fetal distress use STAN (ST analysis) or micro blood analysis

In case of non-progressive labor first stage:

Rupture membranes

Urinary catheterization

Use of pain medication, preferably epidural analgesia

Adequate contractions or augmentation of labor

In case of non-progressive labor second stage in nulliparous women:

Active pushing is recommended

Adequate contractions are recommended

Consider vacuum extraction is the head is < 1/5^th^ palpable per abdomen

Use of partogram

Involvement of the consultant obstetrician in decision making for CS

#

3 Offer external cephalic version in case of non-cephalic position

Use of internal audit on CS care

For each recommendation:

What is your experience in general practice considering this recommendation?

Is general practice according to the recommendation?

If so, what are possible facilitators? What might help others to adhere to these recommendations?

If not, what are the perceived barriers? What might improve care?

Can you provide an example? Can you describe the situation? Do others recognise the situation?

Conclusion and final remarks.
